# Supplementary material for: Multi‐dataset identification of innovative feature genes and molecular mechanisms in keratoconus
Source: J Cell Mol Med. 2024 Sep 19;28(18):e70079. doi: 10.1111/jcmm.70079 (PMC11412914; doi:10.1111/jcmm.70079)
Supplement: Supplementary file 2 — Material S2. [file JCMM-28-e70079-s002.zip › 7.GSEA analysis/5.RNF217-AS1/KEGG_MATURITY_ONSET_DIABETES_OF_THE_YOUNG.html]

Details for gene set KEGG\_MATURITY\_ONSET\_DIABETES\_OF\_THE\_YOUNG[GSEA]

|  || Dataset | RNF217-AS1.group.cls#h\_versus\_l |
| Phenotype | group.cls#h\_versus\_l |
| Upregulated in class | h |
| GeneSet | KEGG\_MATURITY\_ONSET\_DIABETES\_OF\_THE\_YOUNG |
| Enrichment Score (ES) | 0.29332533 |
| Normalized Enrichment Score (NES) | 0.7651477 |
| Nominal p-value | 0.83820665 |
| FDR q-value | 1.0 |
| FWER p-Value | 1.0 |
Table: GSEA Results Summary

  

Fig 1: Enrichment plot: KEGG\_MATURITY\_ONSET\_DIABETES\_OF\_THE\_YOUNG      
 Profile of the Running ES Score & Positions of GeneSet Members on the Rank Ordered List

  

| SYMBOL | TITLE | RANK IN GENE LIST | RANK METRIC SCORE | RUNNING ES | CORE ENRICHMENT || 1 | NKX6-1 | na | 230 | 0.630 | 0.1474 | Yes |
| 2 | ONECUT1 | na | 2266 | 0.329 | 0.1693 | Yes |
| 3 | FOXA2 | na | 2808 | 0.300 | 0.2270 | Yes |
| 4 | HHEX | na | 2984 | 0.292 | 0.2933 | Yes |
| 5 | IAPP | na | 5556 | 0.198 | 0.2676 | No |
| 6 | BHLHA15 | na | 7134 | 0.149 | 0.2586 | No |
| 7 | PDX1 | na | 8658 | 0.113 | 0.2423 | No |
| 8 | PAX4 | na | 9865 | 0.089 | 0.2292 | No |
| 9 | FOXA3 | na | 13865 | 0.032 | 0.1218 | No |
| 10 | HES1 | na | 14513 | 0.025 | 0.1092 | No |
| 11 | NEUROG3 | na | 14797 | 0.021 | 0.1062 | No |
| 12 | NKX2-2 | na | 15054 | 0.018 | 0.1033 | No |
| 13 | PAX6 | na | 15933 | 0.009 | 0.0803 | No |
| 14 | PKLR | na | 16050 | 0.008 | 0.0790 | No |
| 15 | MAFA | na | 18942 | -0.020 | 0.0004 | No |
| 16 | HNF4G | na | 19621 | -0.026 | -0.0126 | No |
| 17 | NEUROD1 | na | 19953 | -0.030 | -0.0149 | No |
| 18 | INS | na | 20462 | -0.035 | -0.0211 | No |
| 19 | GCK | na | 22597 | -0.057 | -0.0687 | No |
| 20 | SLC2A2 | na | 26600 | -0.118 | -0.1551 | No |
| 21 | NR5A2 | na | 27408 | -0.134 | -0.1455 | No |
| 22 | HNF1A | na | 30028 | -0.207 | -0.1703 | No |
| 23 | HNF1B | na | 32103 | -0.297 | -0.1574 | No |
| 24 | HNF4A | na | 33627 | -0.423 | -0.0979 | No |
| 25 | MNX1 | na | 34213 | -0.530 | 0.0148 | No |
Table: GSEA details [plain text format]

  

Fig 2: KEGG\_MATURITY\_ONSET\_DIABETES\_OF\_THE\_YOUNG      
 Blue-Pink O' Gram in the Space of the Analyzed GeneSet

  

Fig 3: KEGG\_MATURITY\_ONSET\_DIABETES\_OF\_THE\_YOUNG: Random ES distribution      
 Gene set null distribution of ES for **KEGG\_MATURITY\_ONSET\_DIABETES\_OF\_THE\_YOUNG**
